# Case Report: A Rare Case of Acute Anterior Myocardial Infarction Simultaneously Associated With Aortic Mural Thrombosis Due to Essential Thrombocytosis

**DOI:** 10.3389/fcvm.2022.840906

**Published:** 2022-02-23

**Authors:** Sheng Ye, Wu-jie Xia, Peng Chen

**Affiliations:** Department of Cardiology, The Second Affiliated Hospital and Yuying Children's Hospital of Wenzhou Medical University, Wenzhou, China

**Keywords:** acute myocardial infarction, aortic mural thrombosis, essential thrombocytosis, percutaneous coronary intervention, anticoagulation

## Abstract

**Background:**

Essential thrombocytosis (ET) simultaneously complicated with acute myocardial infarction and aortic thrombosis is extremely rare and associated with poor outcomes.

**Case:**

A 54-year-old female was admitted to our emergency department with abdominal pain for 3 h. ST-segment elevation in leads V1–V3 on electrocardiography led to the diagnosis of acute anterior myocardial infarction. Coronary angiography demonstrated total occlusion of the proximal left anterior descending artery, and the patient was treated with angioplasty and placement of a drug-eluting stent. CT angiography revealed a massive mural thrombus located in the descending aorta. Bone marrow biopsy confirmed the diagnosis of ET. The patient was successfully treated with antithrombotic therapy and hydroxyurea.

**Conclusion:**

At present, the clinical diagnosis and treatment of ET complicated with acute myocardial infarction and aortic thrombosis are mostly based on literature reports. Early target vessel revascularization, antiplatelet and anticoagulant combined with cytoreductive therapy may improve the prognosis. Clinicians should consider the risk of bleeding and thrombosis and create individualized treatment strategies for these patients.

## Introduction

Essential thrombocytosis is a myeloproliferative disease that often leads to arterial thrombosis (1, 2). Essential thrombocytosis (ET) complicated with acute myocardial infarction (AMI) and aortic thrombosis is extremely rare and is therefore usually undiagnosed. Nevertheless, it is a life-threatening condition that requires emergency treatment. There is no standardized treatment strategy for the condition, although it is often treated with antithrombotic agents and hydroxyurea.

In this study, we reported a case of acute anterior myocardial infarction associated with aortic mural thrombosis due to ET. We successfully treated the patient with percutaneous coronary intervention combined with pharmacologic management.

## Case

**Figure 1:**
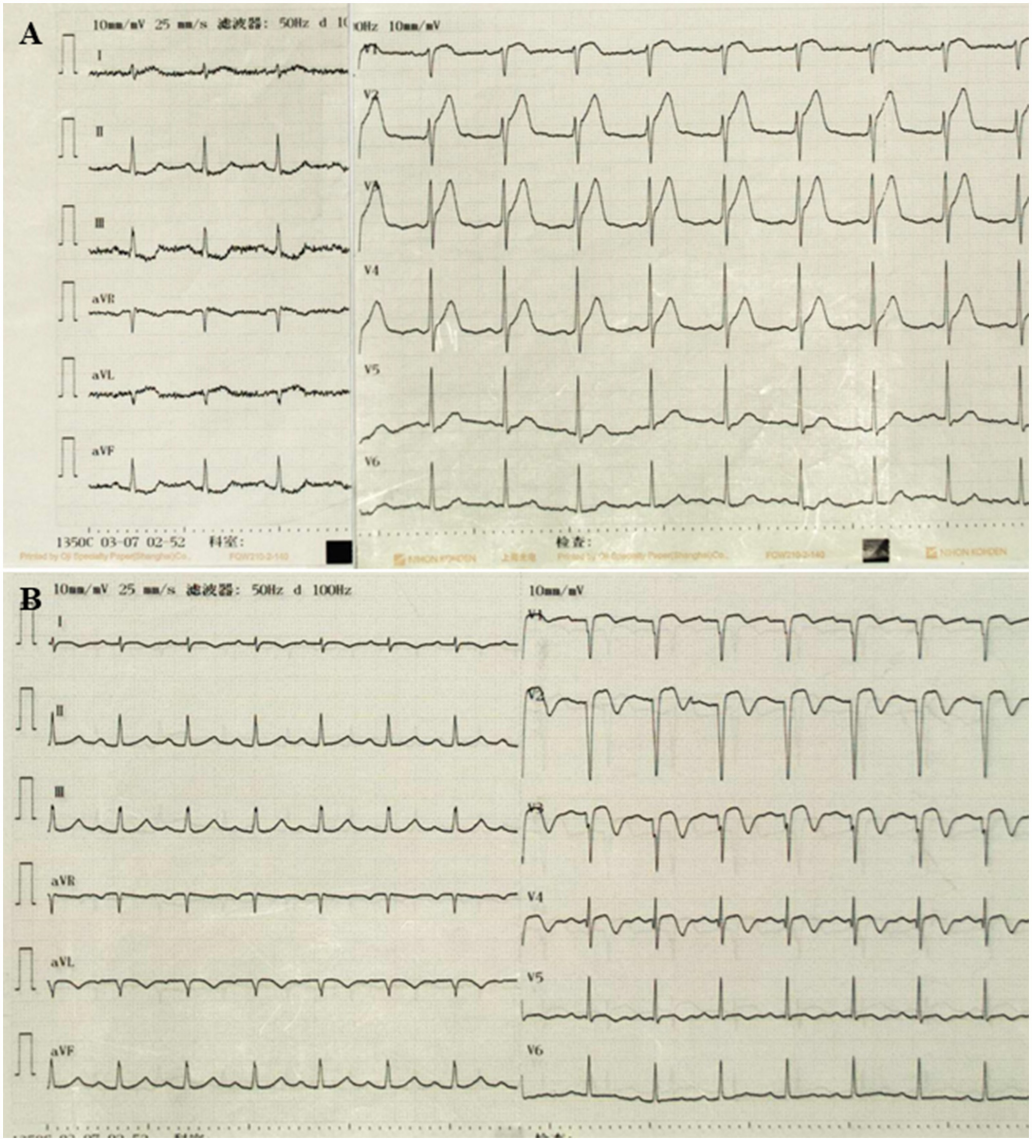
Twelve-lead ECG findings on admission and discharge. **(A)** Admission. **(B)** Discharge.

**Figure 2:**
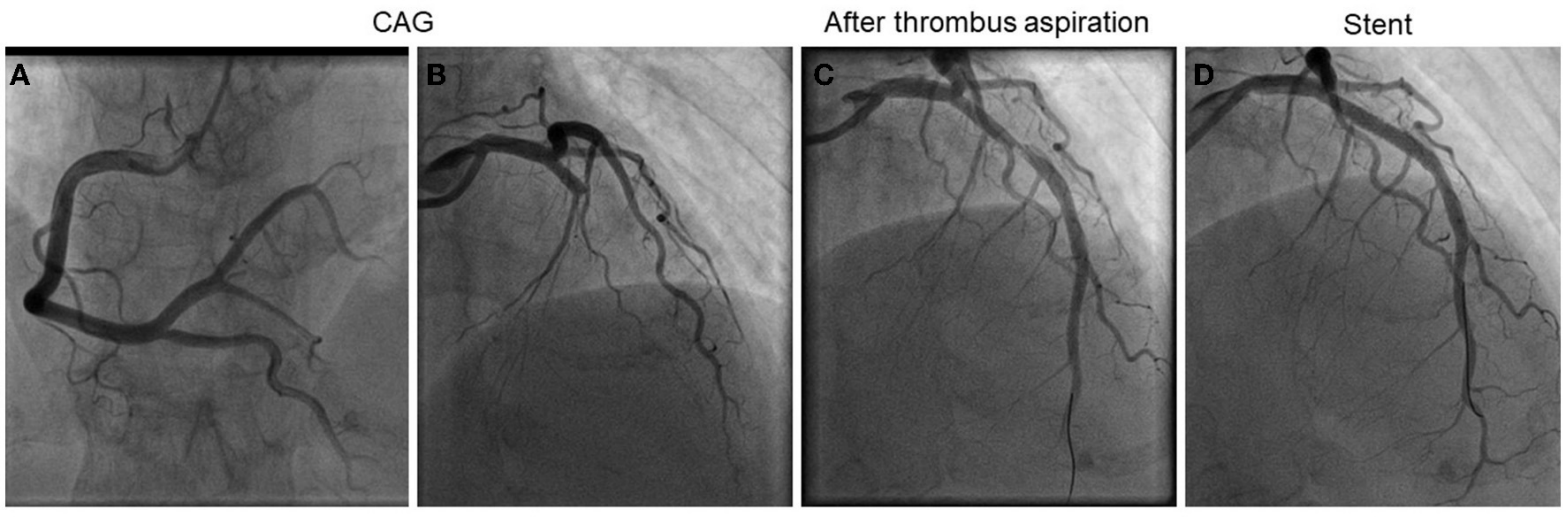
Emergency coronary angiography and percutaneous coronary intervention. **(A)** Right coronary angiography. **(B)** Left coronary angiography. **(C)** After thrombus aspiration. **(D)** After stent.

**Figure 3:**
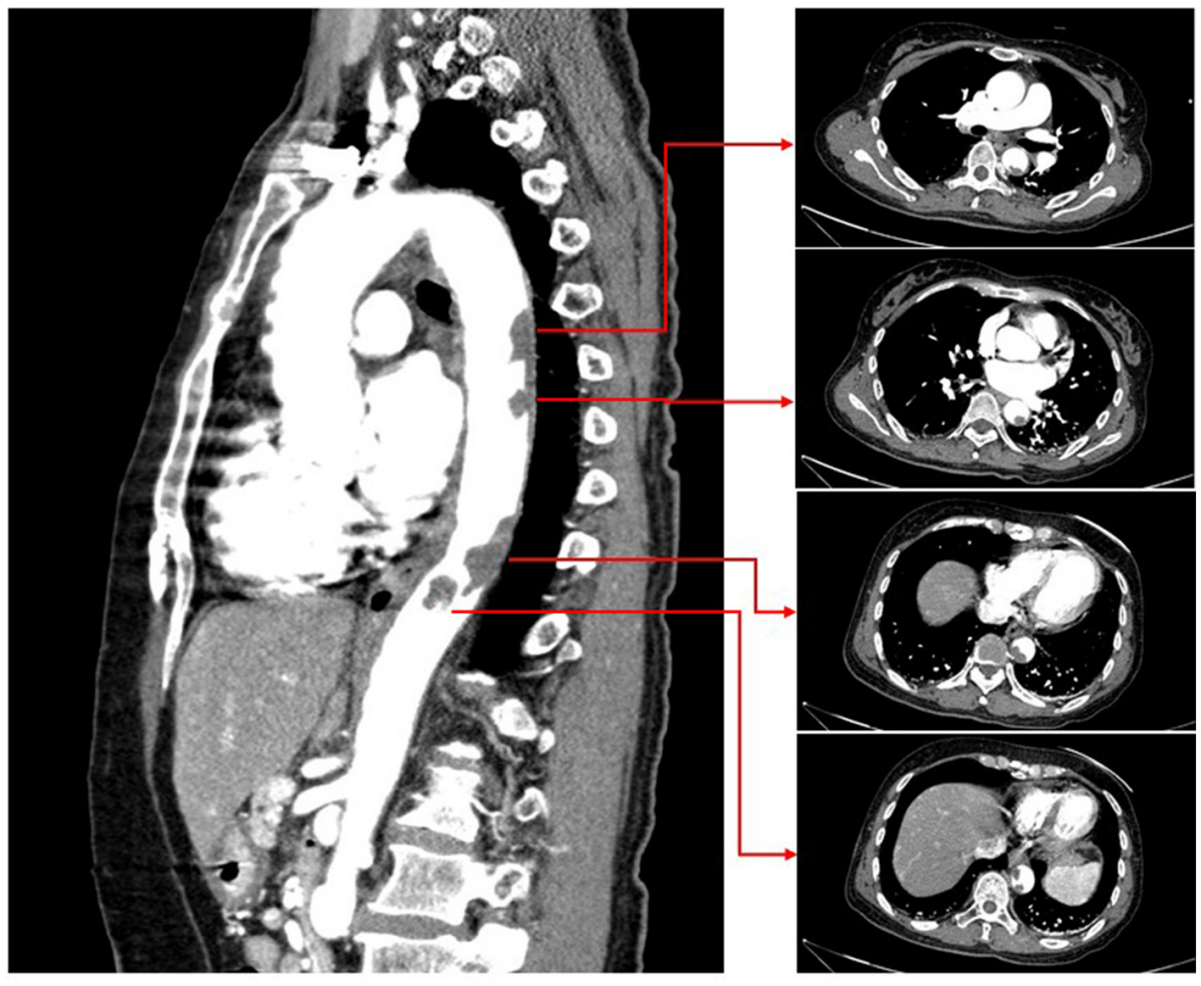
CT angiography revealing massive mural thrombus in the descending aorta.

A 54-year-old female was admitted to our emergency department with abdominal pain for 3 h. Her physical examination was unremarkable, and her vital signs were stable. The patient weighed 51 kg. Laboratory tests revealed a platelet count of 494 x 10^9^/L (normal range 100–300 x 10^9^/L). The troponin-I level was 0.132 ng/ml (normal range 0–0.034 ng/ml), and the D-dimer was 8 μg/ml (normal range 0–0.5 μg/ml). A 12-lead ECG revealed V1–V3 ST-segment elevation (Figure 1A). Acute anterior myocardial infarction was diagnosed, and the catheterization laboratory was engaged. Urgent coronary angiography revealed total occlusion of the proximal left anterior descending artery with thrombus (Figure 2). After thrombus aspiration, the patient was treated with angioplasty and placement of a drug-eluting stent (3 × 18 mm). The patient received unfractionated heparin (UFH) (6,000 IU) during percutaneous coronary intervention. Dual antiplatelet therapy was administrated, including aspirin (100 mg/day) and clopidogrel (75 mg/day). After the immediate percutaneous coronary intervention, the patient continued to complain of recurrent abdominal pain. Laboratory tests revealed a platelet count of 679 × 10^9^/L (normal range 100–300 × 10^9^/L), a troponin-I level of 42.1 ng/ml (normal range 0–0.034 ng/ml), and D-dimer 6.5 μg/ml (normal range 0–0.5 μg/ml). The CT angiography (CTA) revealed a massive mural thrombus in the descending aorta (Figure 3). The patient was started on enoxaparin (8,000 IU/day).

**Figure 4:**
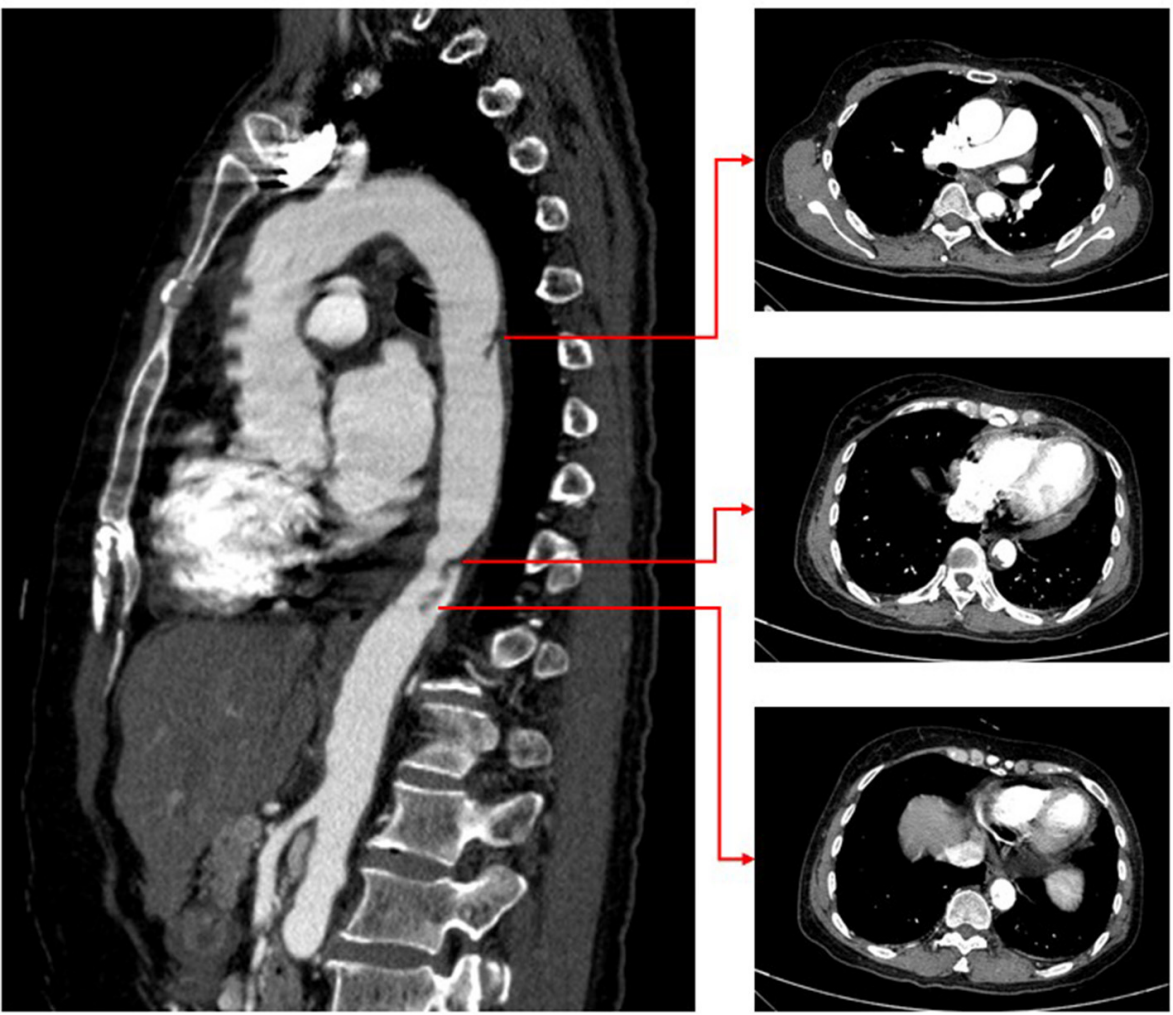
Repeat CT angiography revealing significantly smaller aortic thrombus.

A bone marrow biopsy was performed based on the significantly elevated platelet count, and JAK2 V617F (the most prevalent mutation in ET) was detected. These results were consistent with the diagnosis of ET. The patient denied a family history of myeloproliferative disease. The medications were modified to hydroxycarbamide (1,000 mg/day), enoxaparin (8,000 IU/day), aspirin (100 mg/day), and clopidogrel (75 mg/day). After 10 days of anticoagulation therapy, repeat CTA revealed that the size of the aortic mural thrombus had significantly decreased (Figure 4). The patient received another 10 days of anticoagulation therapy in the hospital. Laboratory tests revealed a platelet count of 299 × 10^9^/L (normal range 100–300 × 10^9^/L), a troponin-I level of 0.032 ng/ml (normal range 0–0.034 ng/ml), and D-dimer 0.25 μg/ ml (normal range 0–0.5 μg/ml). Based on the D-dimer level, which is the most important laboratory indicator of thrombosis, having decreased to the normal range, we assumed that the thrombus had dissolved. However, she refused to undergo another CTA because of economic reasons. Considering the high risk of bleeding associated with triple antithrombotic therapy, anticoagulation was discontinued. The patient was ulti-mately discharged in stable clinical condition. The medications at discharge were modified to hydroxycarbamide (1,000 mg/day), aspirin (100 mg/day), and clopidogrel (75 mg/day). The ECG on discharge revealed Q wave in V1-V3 with T wave inversion (Figure 1B).

## Discussion

Essential thrombocytosis (ET) is a myeloproliferative neoplasm, primarily the megakaryocytic system. It is characterized by a persistent increase in peripheral platelet levels. Hemorrhagic or thrombotic features are the primary clinical manifestations. The annual incidence of ET is about 0.3%. Thrombotic complications are the primary cause of death. However, when platelet levels are extremely elevated (> 1,500 × 10^9^/L), the risk of bleeding increases.

Essential thrombocytosis (ET) that is complicated with AMI is rare (3, 4). Currently, the underlying pathogenesis is unclear, although it may be related to increased platelet count and aggregation. According to the literature, the left anterior descending artery is the most commonly obstructed site, although obstruction can also occur in the right coronary artery (5). At present, there is no standardized therapeutic strategy for acute myocardial infarction complicated with ET. Obstructions are mostly platelet thrombi in these patients, and there may be no atherosclerotic plaques in the coronary arteries. Thrombus aspiration can be used for treatment without coronary stent implantation. However, stents should be considered for patients with evident stenosis in the coronary artery after thrombus aspiration. In our case, because of the severe thrombus burden and stenosis after repeated thrombus aspiration, the patient was treated with angioplasty and placement of a drug-eluting stent.

Aortic mural thrombosis due to ET is rarer (6). There are no appropriate guidelines for managing ET associated with aortic thrombosis. The choice is between pharmacologic and surgical therapy (7). Pharmacologic therapy for ET includes anticoagulation therapy and cytoreductive therapy with hydroxyurea (8). On the other hand, surgical therapy for aortic thrombosis is associated with high mortality and risk of complications and involves bleeding, cerebral and intestinal ischemia, and acute renal failure. Our patient suffered an AMI simultaneously associated with aortic thrombosis due to ET. Because we had previously implanted a drug-eluting stent in the left anterior descending artery, she required dual antiplatelet therapy. If we chose acute surgical treatment, the risk of bleeding would be extremely high. Therefore, we finally chose pharmacologic therapy. Fortunately, after treatment with aspirin, clopidogrel, enoxaparin, and hydroxyurea, the patient's condition stabilized, and repeat CTA revealed a significantly smaller aortic thrombus.

The primary therapeutic goals for ET include preventing thrombosis recurrence. Studies showed that oral anticoagulation (VKA or DOACs) combined with cytoreduction might provide a lower risk of recurrent thrombosis in patients with ET and VTE (9). However, the optimal antithrombotic strategy and duration for AMI and aortic mural thrombosis patients remain unclear. Based on the decreased size of aortic thrombus and D-dimer level in our case, triple antithrombotic therapy turned out to be effective. However, long-term triple antithrombotic therapy may increase bleeding risk. Because ET is a platelet disorder, antiplatelet therapy may be more critical for thromboembolic risk in ET with arterial thrombosis (10). At discharge, this patient's medications included dual antiplatelet therapy (aspirin and clopidogrel) for 12 months as well as cytoreductive therapy. Because the patient refused to undergo another CTA (for economic reasons), we asked her to present for follow-up platelet counts and D-dimer level to monitor thromboembolic risk.

In conclusion, we described a patient with ET who developed a rare combination of AMI and aortic mural thrombosis. This case teaches the importance of medical therapy when patients are not candidates for urgent surgery.

## Data Availability Statement

The original contributions presented in the study are included in the article/supplementary material, further inquiries can be directed to the corresponding author.

## Ethics Statement

Written informed consent was obtained from the individual(s) for the publication of any potentially identifiable images or data included in this article.

## Author Contributions

SY and PC drafted the manuscript and contributed to the case collection. W-jX provided figures and formalized the manuscript. PC reviewed the drafts and approved the final manuscript as submitted. All authors approved the submitted version.

## Funding

The research was funded by Grant 81901409 from the Youth Program of the National Natural Science Foundation of China and Grant Y2020015 from Wenzhou Municipal Science and Technology Bureau.

## Conflict of Interest

The authors declare that the research was conducted in the absence of any commercial or financial relationships that could be construed as a potential conflict of interest.

## Publisher's Note

All claims expressed in this article are solely those of the authors and do not necessarily represent those of their affiliated organizations, or those of the publisher, the editors and the reviewers. Any product that may be evaluated in this article, or claim that may be made by its manufacturer, is not guaranteed or endorsed by the publisher.
